# The effectiveness of malaria camps as part of the Durgama Anchalare Malaria Nirakaran (DAMaN) program in Odisha, India: study protocol for a cluster-assigned quasi-experimental study

**DOI:** 10.1080/16549716.2021.1886458

**Published:** 2021-04-18

**Authors:** Danielle C. Ompad, Anne Kessler, Anna Maria Van Eijk, Timir K. Padhan, Mohammed A. Haque, Steven A. Sullivan, Yesim Tozan, Joacim Rocklöv, Sanjib Mohanty, Madan M. Pradhan, Praveen K. Sahu, Jane M. Carlton

**Affiliations:** a School of Global Public Health, New York University, New York, NY, USA; b Center for Genomics and Systems Biology, Department of Biology, New York University, New York, NY, USA; c Department of Molecular & Infectious Diseases, Community Welfare Society Hospital, Rourkela, Odisha, India; d Department of Public Health and Clinical Medicine, Umea University, Umea, Sweden; e Department of Health & Family Welfare, State Vector Borne Disease Control Programme, Bhubaneswar, Odisha, India

**Keywords:** Malaria camp, malaria, screening and treatment, plasmodium, malaria control program, Odisha, DAMaN, cluster-assigned quasi-experimental study

## Abstract

The Indian state of Odisha has a longstanding battle with forest malaria. Many remote and rural villages have poor access to health care, a problem that is exacerbated during the rainy season when malaria transmission is at its peak. Approximately 62% of the rural population consists of tribal groups who are among the communities most negatively impacted by malaria. To address the persistently high rates of malaria in these remote regions, the Odisha State Malaria Control Program introduced ‘malaria camps’ in 2017 where teams of health workers visit villages to educate the population, enhance vector control methods, and perform village-wide screening and treatment. Malaria rates declined statewide, particularly in forested areas, following the introduction of the malaria camps, but the impact of the intervention is yet to be externally evaluated. This study protocol describes a cluster-assigned quasi-experimental stepped-wedge study with a pretest-posttest control group design that evaluates if malaria camps reduce the prevalence of malaria, compared to control villages which receive the usual malaria control interventions (e.g. IRS, ITNs), as detected by PCR.

## Background

Despite declines over the past decade, malaria remains an important global public health problem in India. India is one of twenty countries accounting for 85% of all malaria cases and deaths in 2018 and 47% of all *Plasmodium vivax* cases globally [1]. Of note, between 2017 and 2018 there was a 27% reduction in reported malaria cases, a margin of 2.6 million cases [1]. The eastern Indian state of Odisha accounted for 40.9% of cases in 2016, 41.2% in 2017, 15.4% in 2018, 11.8% in 2019, and 27.8% in 2020 (through February) [2], despite comprising only 4.9% of the nation’s land area [3] and 3.5% of its population [4].

Odisha is known for its hilly, forested, and inaccessible regions, and for the indigenous ‘tribal’ people who live there. (Although the global term ‘indigenous peoples’ is widely used in the literature, it is a debated term in India [5]. The Indian government uses the term ‘tribal’ or the constitutionally-recognized category of ‘Scheduled Tribes**’** to refer to these communities [6].) Tribal communities in India are particularly prone to malaria due to geographical marginalization, poor access to health care, low socioeconomic status, and other social factors including cultural and religious values and beliefs [7,8]. Odisha is divided into 30 districts [9], 314 blocks, and 53,845 villages [10]. The climate is subtropical, with four seasons: winter (January–February), pre-monsoon (March–May), southwest monsoon (June–September), and northeast monsoon (October–December) [3]. More than 47,000 Accredited Social Health Activists (ASHAs; community health workers) are involved in malaria control activities [11,12].

Malaria transmission occurs predominantly in forested and forest fringe regions in Odisha, with many communities experiencing poor access to health care, especially during the rainy season. *Plasmodium falciparum* is the major parasite species (>90% of cases), and the major malaria vectors are *Anopheles fluviatilis* found in inaccessible, hilly, and forested regions [13,14], and *Anopheles culicifacies* sp. C and E in plains villages where they breed in rice fields [15,16]. Even on a relatively small spatial scale (e.g. a village), malaria transmission is heterogeneous, with a subset of people experiencing greater rates of mosquito biting [17]. Distribution of insecticide-treated nets (ITNs) in areas with an annual parasite incidence (API; cases per 1,000 people) ≥2 forms an important part of malaria control in the state, and indoor residual spraying (IRS) with dichlorodiphenyltrichloroethane (DDT) is offered twice a year in high-risk malarious areas. In 2016, 58.3% of blocks had an API ≥2, with 34.4% ≥10.

Antimalarial drug resistance has been reported in *P. falciparum* infections at various sites in Odisha, primarily through molecular studies that sequence the parasite genes known to be involved. For example, mutations in *P. falciparum* genes associated with chloroquine resistance [18–21] and sulphadoxine-pyrimethamine resistance [19,20] have been identified throughout the state. Routine molecular surveillance of parasites in these areas has also identified changes in the frequency of drug resistant mutations as different antimalarial drugs have been discontinued, such as the reversion to sensitive genotypes due to removal of the drug chloroquine [19]. More recently, sequencing studies have been used to identify if mutations in the *P. falciparum* kelch13 gene, implicated in resistance to artemisinin, have arisen in multiple parts of Odisha [19,22], but as yet no such mutations have been identified.

The persistently high malaria burden in remote forested areas of Odisha has led to the introduction of ‘malaria camps’ (MCs) by the Odisha State Malaria Control Program (MCP), where teams of health workers visit villages to educate the population, enhance vector control with long-lasting insecticide-treated nets (LLINs) and IRS, and perform village-wide screening with rapid diagnostic tests (RDTs) and treatment for malaria. The MCs are part of a program, Durgama Anchalare Malaria Nirakaran (DAMaN) ‘malaria control in inaccessible areas,’ and have two main components, mass screening and treatment of all malaria positive cases and simultaneous intensive vector control using LLIN and IRS, allowing for both the parasite and mosquito to be targeted in parallel. The camps appear to be effective, but this has been hard to assess in the context of ongoing changes such as LLIN introduction. The goal of the study protocol described here is to evaluate the effectiveness of MCs on malaria prevalence.

## Methods/design

### Aims and objectives

The goal of this protocol is to evaluate the effectiveness of malaria camps (MCs) by determining if they reduce malaria and to characterize malaria transmission in intervention villages over time. Specifically, this pilot study is designed to demonstrate proof-of-concept and feasibility, and aims to assess if MCs in intervention villages reduce the prevalence of malaria compared to standard malaria control efforts in control villages. In addition, the study aims to investigate the out-of-pocket costs and impact of malaria from the patient’s perspective in these hard-to-reach tribal communities.

Secondary objectives of this study include: determining if MCs reduce the prevalence of subpatent, symptomatic, and asymptomatic malaria as detected in the study surveys as well as in local clinics (e.g. passive case data collected through existing surveillance networks; symptomatic malaria prevalence only); assessing malaria exposure, immunity, and risk among participants in each arm of the study through high-throughput detection of antibodies; assessing the genetic epidemiology (i.e. species, genetic diversity, clonality, drug resistance) of *Plasmodium* parasites in each study arm using molecular and genomic methods; characterizing the social factors affecting the implementation of MCs using interviews; surveying mosquito vectors in and around the houses of subjects enrolled in each arm; evaluating the impact of the interventions using epidemiological compartmental modeling of host-vector interactions; and building research capacity in Odisha and India to help train the next generation of malaria and mosquito vector biologists.

### Study design

The effectiveness of the MC intervention is being evaluated with a quasi-experimental cluster-assigned stepped-wedge study with a pretest-posttest control group design; clusters (i.e. villages) are not randomized to the intervention. The main aim of the study is to determine if MCs reduce the prevalence of *Plasmodium* infection as detected by PCR. At baseline, villages are assigned to one of three study arms. In year 1, Arm A villages receive MCs for the first time; Arm B villages serve as control villages where no MCs but routine malaria control activities are implemented; and Arm C are villages that have already had at least one round of MCs prior to initiation of this study that continue to receive the MCs, providing an opportunity to study the longer term effects of the MCs. In the second year, both Arm A and Arm B villages receive the intervention (i.e. a non-randomized stepped-wedge design). MC effectiveness is evaluated from epidemiologic surveys and PCR detection of malaria prevalence in villages with MCs as compared to those without MCs.

This proof-of-concept pilot study was designed in part to build and test the relationship with the Odisha Malaria Control Program (MCP) in the Ministry of Health. Because of the relatively short timeline from funding to field implementation, the districts needed to make decisions on where the malaria camps were going to be implemented rather quickly. While randomization was not possible at that time, the MCP did balance the assignment of villages with respect to ecology. The stepped wedge design was chosen for the same logistical restraints that resulted in the decision to not randomize the villages for this pilot study.

### Description of intervention

The MCs implemented by the Odisha State MCP in remote forested areas of the state involve teams of health workers visiting villages to educate the population, enhance vector control with LLINs and indoor residual spraying (IRS), and perform village-wide testing with rapid diagnostic tests and treatment for malaria. The MCP has dramatically scaled up efforts to prevent, diagnose, and treat malaria under the DAMaN program, with apparently rapid and impressive results [23]. The first-line treatment for uncomplicated *P. falciparum* as per Indian national guidelines is artesunate 4 mg/kg body weight per day (3 days); sulfadoxine-pyrimethamine (SP) 25 and 1.25 mg/kg body weight, respectively (one dose) on day 1; and primaquine 0.75 mg/kg body weight or 45 mg (one dose) on day two. Similarly, the first-line treatment for *P. vivax* is chloroquine (CQ) 10 mg/kg body weight (day 1 and day 2) and 5 mg/kg body weight (day 3), along with primaquine 0.25 mg/kg body weight (one dose) for 14 days.

The program consists of two and up to three MC rounds administered per annum. The first round (round 1) during the pre-monsoon season includes testing for the whole village population with treatment of RDT+ individuals along with LLIN distribution, IRS, and intensified campaigning with ASHAs. The second round (post-monsoon season, round 2) screens only febrile adults but all children (<5 yr age) and lactating/pregnant mothers regardless of fever status. This is because round 1 is expected to reduce malaria by >90%, and subsequent rounds need only target the remaining febrile cases or potentially asymptomatic cases that have become symptomatic in the interim. The necessity of round 3 MCs is determined based on the coverage and success of rounds 1 and 2 and is, therefore, optional.

For each MC round, the DAMaN team is in each village for one to three days, depending on the size of the village. The MCs also offer a range of malaria awareness activities such as plays containing malaria prevention messages performed by traveling troupes, bednet use ‘performances’ in government high schools with hostels, and vans with loudspeakers that play popular local songs that incorporate malaria prevention guidance in the lyrics and have mosquito control messages displayed on their sides. In addition, the MCs include nutritional screening of children and pregnant women.

### Target population

**Figure 1.**
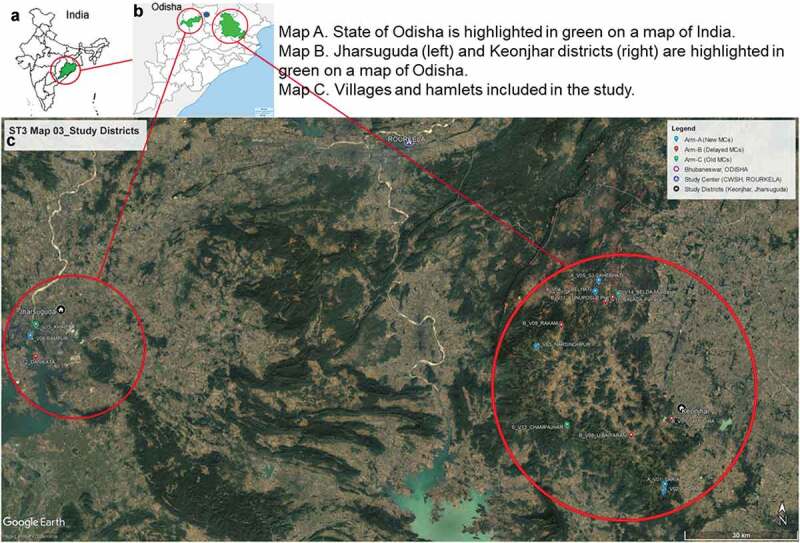
Maps of the study districts

The eastern state of Odisha is a predominantly (83%) rural state of approximately 42 million people [4] with ~62% of the rural population consisting of tribal populations, altogether representing >60 different ethnic communities. These tribes inhabit the hilly ranges including in Jharsuguda and Keonjhar districts [24] where the study is located (see Figure 1 for a map of the region). The tribal population makes up to about 30.5% of the total district population in Jharsuguda and 45.5% in Keonjhar [26]. Major tribes in the two districts include Bathudi, Bhuyan, Bhumij, Gond, Ho, Juang, Kharwar, Kisan, Kolha, Kora, Munda, Oraon, Santal, Saora, Shabar and Sounti [27]. The tribal populations in Odisha experience substantial socioeconomic disadvantage, as evidenced by low literacy rates (52.2% overall); underemployment (35% of workers aged 15 years & above worked for 12 months in the last year); few homes with electricity or solar power (15.6 and 0.6%, respectively) as their main lighting source, no latrine on premises (92.9%), or a water source inside (6.2%); and high prevalence of open defecation (92.9%) [28]. Agriculture, mining, quarrying, and household industries are the main types of employment [25].

A recent study in the adjacent Sundargarh district found that behavioral risk factors for malaria include being outside in the evening (34.5% among adult men and 7.9% among adult women aged 15+ years and 0.7% among children) and sleeping outside (7.5% vs. 0.5% and 0%, respectively, p < 0.001). Although women were more likely to get up before 6 a.m. (86.6%, vs. 70.5% among men, 50.7% among children, p < 0.001), men were more likely to be outside in the early morning (77.6% among men, 11.2% among women, and 11.1% among children, p < 0.001). More children used insecticide treated nets the previous night (73.4%) than men (45.6%) or women (39.6%), and repellents were used by 29.5% of 234 households (insecticide creams were not used at all) [29].

Tribal areas such as the Jharsuguda and Keonjhar districts, where this study is situated, are the most seriously afflicted with malaria, displaying meso- to hyperendemic transmission. IRS coverage in malarious regions is approximately 90% and the last ITN distribution was in July/August 2017 [30]. These communities are our target population.

Village residents are eligible for the study if they are (1) aged 12 months to 69 years and (2) have an apparent complete understanding of the study procedures as outlined in the consent form. There are no exclusion criteria based on residency length, pregnancy, social class, or health conditions.

### Sample size calculations

Statistical significance is not the central goal of this two-year pilot study. The sample size was estimated based on the stated requirement of a minimum of 2,000 participants in the National Institutes of Health request for proposals in concert with study logistics [31]. We used Stata 16.0 to calculate power using the *steppedwedge* command [32] which is used to calculate power for stepped wedge cluster-randomized controlled trials. We note that this trial is not randomized (i.e. it is cluster-assigned); as a result, power estimation should be interpreted as a guide.

An average village in the malarious areas of Odisha has ~250 (range: 60–850) households and 1,200 (range: 300–4,000) people, and we aim to enroll 163 people per cluster. In our previous studies, *Plasmodium* infection prevalence by PCR in the forest villages around Rourkela was 16% during the rainy season [33], and we consider a difference of 6% (e.g. from 16% to 10%) in *Plasmodium* infection prevalence by PCR after two rounds of MCs between control and intervention clusters measured during the rainy season in the second year, to be a meaningful reduction. For our sample size calculations, we compared between clusters in arm A (intervention) and arm B (control), and used an intracluster correlation coefficient (ICC) of 0.02 (although studies reporting an ICC for a malaria outcome in India could not be found, a study on maternal and neonatal health in India showed that this number may be a conservative estimate compared to outcomes with a similar low prevalence [34]).

We calculated power for the stepped wedge design using Arms A and B; Arm C was not included in the power calculations because we do not have pre-intervention data for that arm. Two study arms of 5 clusters each and an average cluster size of 163 (total sample size 1,630, 1:1 sample size ratio) allows us to detect a reduction from 16.0 to 10.0% prevalence of *Plasmodium* infection by PCR with 80% power and 5% significance level; we use a 15% higher sample size (~187 per cluster, n = 1,870) to allow for loss- to - follow - up.

Revised sample size: Based on our experiences recruiting in the field in August and September 2019, we increased the sample size from 2244 to 2700 and add two new clusters (villages). To achieve the sample size of 2700, we are enrolling one additional village in each arm for a total of six villages each in Arms A and B and three in Arm C (for a total of 15 villages). Six clusters each in Arms A and B and an average cluster size of 163 (total sample size 1,956, 1:1 sample size ratio) allows us to detect a reduction from 16.0 to 10.5% prevalence of *Plasmodium* infection by PCR with 80% power and 5% significance level; we use a 18% higher sample size (~193 per cluster, n = 2,316) to allow for loss-to-follow-up. The increase in sample size allows us to maintain study power, and the rationale for the increase is twofold. First, two of the villages were inhabited by the Juang People, one of the most geographically and culturally isolated tribal groups specific to the Keonjhar district [35]. Willingness to participate among this group was low, and we anticipate high loss-to-follow up rates from individuals in this tribe despite giving consent. Second, study staff noticed in most villages that some participants discarded their study cards. These study cards are labeled with the study ID number with no personal identifiers and are the way in which we track participants over time. As a result, we are concerned that follow-up rates will be lower than we first anticipated across all villages.

### Sampling of villages and assignment to study arms

We selected one or more district blocks with a high API (>2) from the study districts. Villages/hamlets are the units of the MCs and are therefore the clusters in this study. We assign the villages/hamlets into two study arms: Arm A ‘new-MC’ (n = 6 villages that start MCs in year 1) and Arm B ‘delayed-MC’ (n = 6 villages with routine malaria control activities in year 1 and MC in year 2). A third study arm (Arm C ‘old-MC’) is comprised of n = 3 villages where MCs have already been implemented, providing an opportunity to assess the effects of the MCs over a longer period of time. After assignment to study arms, a cohort is enrolled during a baseline visit in all the clusters. In the old-MC clusters (where MCs have occurred) and in the clusters not receiving MCs, malaria activities continue as planned by the MCP. In the new-MC clusters, the MC intervention is introduced (one round of screening and treatment for the whole cluster population pre-monsoon, followed by at least one round of screening of adult fever cases, children under 5 years, and pregnant women only post-monsoon). The exact villages and districts were chosen in consultation with the Odisha MCP which balanced the assignments in terms of ecotype (e.g. forest, mining, river, plain, etc.). Because this is a community-based intervention, participants are not blinded.

### Recruitment

To recruit participants, a field team travels to the villages at the same time when the DAMaN team delivers the intervention. Within each village, the DAMaN team sets up their screening and treatment camps in a central location. Our team sets up their operations adjacent to the DAMaN team. As each village resident completes their DAMaN visit, they are informed of the nature and purpose of the study and invited to enroll.

### Consent

For those who express interest in the study, field staff read aloud a consent form in one of the local languages (i.e. Oriya or Hindi). Eligible participants are offered a copy of the consent form in the local language to read along with the field staff member. Persons aged 18 years or older who are mentally capable of consenting are provided a consent form to review and sign. For children under seven years of age, a consent form signed by a parent or guardian is required. For children between seven and 17 years of age, parental consent and an additional child assent is required for participation. If a person cannot write, they are asked to place a thumbprint on the consent form in the presence of a literate witness (i.e. another adult in the household or a neighbor), who also signs the form. Ethical approval for the study was obtained from the institutional review board at New York University (New York, NY, USA) and the institutional ethics committee at Community Welfare Society Hospital (Rourkela, Odisha, India). The study was also approved by the Indian Council of Medical Research Health Ministry’s Screening Committee.

### Data collection

#### Survey

A brief survey is administered to consenting adults and older children (aged 17 and older) via Samsung Galaxy tablets (10.1” 32GB SM-T580 – International Version) loaded with a REDCap (Vanderbilt University, Nashville, TN, USA) mobile application instrument. A parent or guardian provides proxy responses for children aged under 17 years. The survey includes measures of demographics, socioeconomic status (SES), malaria knowledge, malaria prevention, and previous malaria and treatment. With respect to demographics, participants are asked to provide their age, sex, and number of people living in the household; proxy interviewees for children are asked to describe their relationship to the child. SES measures include educational attainment and occupation.

Malaria knowledge focuses on signs and symptoms; respondents are asked open-ended questions and field staff record responses. For malaria prevention, participants are asked if they slept outside and whether they slept under a bednet when they did so. For previous malaria and treatment, participants are asked if they have ever had malaria, when was the last time they had it, whether they sought advice or treatment last time, the time to treatment, whether they were tested for malaria last time, and the result of the last malaria test.

At the time of enrollment, each study participant receives a blue study card with their study participant number. The study card is a means to provide anthropometric and blood testing results to the study participant at each visit and also allows for participant tracking in subsequent visits.

#### Anthropometrics

Field staff measure each participant’s body temperature, height, weight, and mid upper arm circumference (MUAC). Body temperature is measured temporally in degrees Fahrenheit with a non-contact infrared thermometer (Dr. Morepen FS-300, Korea) to the nearest tenth of a degree. Height is measured with a height measuring tape (PMG Eco Series, India) in centimeters. Weight is measured in kilograms with a scale (Dr. Morepen DS-03, India). MUAC is the circumference of the left upper arm, measured at the mid-point between the tip of the shoulder and the tip of the elbow (i.e. olecranon process and the acromion). Participants are asked to remove any clothes that may hinder the measurement (preferable directly on skin, but tight clothes can be accepted if they are difficult to remove). MUAC is measured in centimeters to one decimal using a Myotape/tape measure (Jyoti IND/09/12/309, India).

#### Malaria cost survey

Participants who self-report having malaria in the past eight weeks are administered a malaria cost survey to assess the total out-of-pocket cost of a malaria illness episode in our study setting alongside the trial. The malaria cost survey has questions eliciting information on treatment-seeking behavior and associated out-of-pocket spending, work and school absenteeism, and income loss incurred by participants or their caregivers during the illness episode.

Out-of-pocket costs include direct and indirect costs borne by households during a malaria illness episode. Direct costs include all out-of-pocket medical (consultation fees, tests, medications) and non-medical (transport, meals, lodging) expenditures for seeking and receiving treatment for malaria while indirect costs consist of lost paid work for participants, or, where applicable, their caregivers during malaria illness. We will value lost paid work due to malaria illness as the higher of the self-reported or estimated income loss; the latter is estimated conservatively as the product of the number of reported work-days lost by the patient or their caregivers and the minimum daily wage for Odisha state set by India’s Labour Bureau [36]. We will also report on the number of school days missed due to malaria illness. The economic value of school absences due to malaria illness is estimated as the product of the number of school-days missed due to illness and the daily per-capita cost of providing education in India [37].

The total out-of-pocket cost per malaria episode is obtained by summing direct and indirect costs. If there are missing cost data, depending on the amount and the reasons for the data being missing, we will apply naive or multiple imputation methods to the dataset to derive unbiased and efficient estimates [38]. We will report the mean, standard deviation, median, and interquartile ranges for costs and days of missed school and work lost per malaria episode. Costs will be reported in US dollars (USD) for the initial year of the trial. All costs are collected in local currency, and will be adjusted for inflation [39], if necessary, before conversion to USD [40].

#### Sample collection and processing

In addition to completion of a study questionnaire and anthropometric measurements, study enrollment procedures include a finger prick by sterile lancet for small blood volume sampling (300 ul; Sarstedt microvette CB300 with EDTA), thin and thick blood smears, blood spots (Whatman FTA Blood Stain Cards), and point of care (POC) testing (e.g. hemoglobin [HemoCue America, Brea, CA], malaria rapid diagnostic test [RDT, described below]). All individuals are screened for *Plasmodium* infection by RDT and species-specific PCR. Vacutainers (5 ml, BD ACD) of venous blood are requested from individuals found to be *Plasmodium* positive by RDT. All vacutainers and small volume microvettes are immediately refrigerated on ice and shipped overnight in insulated coolers to the main laboratory where they are processed. Blood components (e.g. red blood cells (RBC) pellet, plasma) are isolated and stored at −80°C until assayed.

#### Rapid diagnostic testing for P. falciparum and P. vivax

Finger prick blood samples from all study subjects are tested for *P. falciparum* and *P. vivax* using the point-of-care (POC) test FalciVax (Zephyr Biomedicals, Verna, Goa, India). FalciVax is a lateral flow RDT that detects species-specific *Plasmodium* antigens in the peripheral blood. Using the sample loop included in each RDT kit, five microliters of blood and two drops of buffer are added to the cartridge. The results for each RDT is read after 20 minutes at room temperature and recorded.

#### Evaluation of Giemsa-stained blood smears

Thin smears are fixed in methanol and dried prior to Giemsa staining alongside thick smears. Stained smears are evaluated quantitatively and qualitatively for asexual (i.e. ring, trophozoite, schizont) and sexual (i.e. gametocytes) stage parasites at the species level by two independent microscopists as previously described [33].

#### Molecular detection of P. falciparum and P. vivax infection by PCR

The RBC pellet isolated from the small blood volume obtained from each study participant is stored at −80°C until assayed. Upon thawing, DNA is extracted using QIAGEN’s QIAamp DNA mini Kit. A final elution volume of 50 ul is used. Molecular detection of *P. falciparum* and*/*or *P. vivax* is determined by PCR amplifcation of multicopy target loci (Pvr47 present as 14 copies in the *P. vivax* genome, and Pfr364 present as 41 copies in the *P. falciparum* genome) as described [41], GoTaq Green Mastermix (Promega), and a Veriti 96-well Fast Thermal Cycler (Applied Biosytems). PCR products are visualized by gel electrophoresis using ethidium bromide and a Gel Doc EZ documentation system (BioRad). Positive and negative controls are included in all experiments, and 10% of samples are assayed at an independent lab for QC purposes.

#### Plasmodium antibody quantification by Luminex MAGPIX

Seventeen recombinant *P. falciparum* and *P. vivax* proteins and/or peptides (Table 1) are used in the multiplexed, bead-based assay. All antigens are coupled to unique Luminex Magplex magnetic microspheres and stored in the dark at 4°C until use.

**Table 1.** *Plasmodium* and control antigens in serology panel

Classification	Antigen	Function	Source and reference
*P. falciparum*	EBA140	IEI	J. Beeson [63]
	EBA175	IEI	J. Beeson [63]
	EBA181	IEI	J. Beeson [63]
	Etramp5.Ag1	IEI (interface between IE and PVM)	K. Tetteh [64]
	HSP40.Ag1	IEI (molecular chaperone)	K. Tetteh [65]
	MSP2 CH150	IEI	Cavanagh [66]
	MSP2 Dd2	IEI	Cavanagh [66]
	PfAMA1	IEI (mediates tight junction formation)	Blackman/Crick [67]
	PfGlurp.R2	IEI (shed from merozoite)	Theisen [68]
	PfMSP1_19_	IEI (mediates initial attachment)	Holder/NIMR/Crick [69]
	Rh2 2030	IEI	J. Beeson [70]
	Rh4.2	IEI	J. Beeson [71]
	Rh5	IEI	Draper/Jenner [72]
*P. vivax*	PvAMA1	IEI (mediates tight junction formation)	C.H. Kocken [73]
	PvMSP8	IEI	K. Tetteh; unpublished
	PvMSP10	IEI	K. Tetteh; unpublished
	PvMSP1_19_	IEI (mediates initial attachment)	T. Holder [74]
*Other*	GST	Control fusion protein	GE Healthcare
	Tet toxoid (TT)	Tetanus vaccine, internal assay control	NIBSC

IEI = Intra-erythrocyte invasion

Plasma pellets isolated from the small blood volume obtained from each study subject are prepared at 1/200 in a buffer of PBS, 0.05% Tween, 0.5% BSA, 0.02% sodium azide, 0.1% casein, 0.5% PVA, 0.5% PVP, and *E. coli* extract. All samples, including controls and blanks, are run in duplicate according to standardized procedures [42]. Plates are analyzed on a Luminex multiplex MAGPIX instrument, and xPONENT software is used for data acquisition (Luminex Corp., Austin, TX). Raw values are determined for all antigens in all samples (raw median fluorescent intensity [MFI_Ag_]) and converted to Net MFI (Net MFI_Ag_ = raw MFI_Ag –_ background MFI_Ag_) where background MFI_Ag_ is the mean MFI of a given antigen in the blank wells. For each antigen, seropositivity is defined as mean Net MFI_negative pool_ plus three standard deviations (SD).

#### Genetic epidemiology of Plasmodium parasites

The *Plasmodium* species composition and complexity of infection for each arm are determined. We use deep sequencing methods [19] to run multiplex amplicon-seq assays that assay (1) *P. falciparum* drug resistance alleles to infer the resistance of circulating parasites to antimalarial drugs, and (2) *P. falciparum* and *P. vivax* highly polymorphic loci for genetic diversity measurements. The latter assay determines if, as transmission decreases, there is a decrease in parasite genetic diversity, as has been shown to occur with *P. falciparum* [43]. We also undertake whole genome sequencing of a limited number of single genotype *P. falciparum* and *P. vivax* isolates to determine their genetic diversity and phylogenetics in comparison to our previously sequenced Indian isolates [44,45].

#### Follow-up

Follow-up visits are conducted immediately prior to the MC camps in each intervention village and during roughly the same time period in the comparison villages. Participant IDs are confirmed via presentation of the individual’s blue study card. Follow-up visit procedures parallel those employed during the baseline enrollment visit, including completion of the study questionnaire, anthropometric measurements, malaria RDT and hemoglobin POC testing, and collection of a small volume of blood by finger prick. Vacutainers (5 ml, BD ACD) of venous blood are requested from individuals found to be *Plasmodium* positive by RDT.

#### Vector survey

In each study village, eight randomly selected households and eight cattle sheds are surveyed for mosquitoes using light trap, hand catch, and pyrethrum space spray collection methods. UV light traps are set for a 12 hour window (6 pm to 6 am) both inside and outside of the selected households. Mosquitoes caught in the traps are identified morphologically to the species level, and mosquito density per trap per night is calculated. Hand catch collections of resting mosquitoes are performed inside each household and cattle shed for 15 minutes via sucking tubes. Hand caught mosquitoes are also identified morphologically to the species level, and per hour species mosquito density is calculated. Pyrethrum spray collection of the resting mosquitoes in one bedroom of each village is performed. The collected mosquitoes are identified morphologically to the species level, and per room density is calculated. Blood meals of the vectors are collected on filter paper to identify their feeding behavior. All mosquito collection data are tabulated manually. The selected households are evaluated using a questionnaire to document features and characteristics of the dwelling and surrounding region relevant for parasite transmission and increased risk of infection. The household data are collected via the REDCap Mobile App on Samsung Galaxy tablets.

### Definition of outcomes

Primary outcome measures are *Plasmodium* presence and species. Parasite presence is defined as PCR-positive for any malaria parasite species, and parasite species are defined as PCR-positive for *P. falciparum* and/or *P. vivax*.

Secondary malaria outcome measures include malaria as detected by RDT, gametocyte density, *Plasmodium*-specific serology, and *Plasmodium* parasite genetic epidemiology. Malaria by RDT is defined as RDT-negative vs. RDT-positive. We further identify asymptomatic and subpatent infections. Asymptomatic infections are defined as PCR and/or RDT positive for any malaria species with absence of documented fever or self-reported fever in the last 48 hours. Subpatent malaria is defined as RDT-negative and PCR-positive. Gametocyte density is measured by quantifying the number of gametocyte infected erythrocytes and dividing by the number of leukocytes. To account for a mixed-infection (either by species or by multiple strains), we take the gametocyte to trophozoite ratio to reduce any apparent bias. *Plasmodium*-specific serology is analyzed as a continuous antibody titer variable as well as a categorical (seropositive vs. seronegative) variable. In terms of *Plasmodium* parasite genomic epidemiology, variables include clonality, genetic diversity, and molecular force of infection.

Secondary anthropometric outcome measures include hemoglobin, body mass index (BMI), body temperature, and MUAC. Hemoglobin is measured by POC testing which provides both continuous and categorical data (when analyzed by local standards) regarding anemia status. BMI is calculated as kilograms/meters^2^ and analyzed as a continuous and categorical variable. BMI categories (i.e. underweight, normal weight, overweight, and obese) are based on adult and child standards for Asian Indians [46–49]. Body temperature is analyzed as a continuous and categorical variable. We have created two categorical variables for temperature, fever (≥ 99.5 °F) and hypothermic (≤ 95.0 °F). MUAC is measured as a continuous and categorical variable indicating malnutrition and based on WHO standards.

### Statistical analyses

*Plasmodium* infection prevalence by PCR at the third follow-up visit is the main outcome and will be compared across Arms A and B, taking clustering into account with generalized estimating equations in Stata version 16.1 or higher (StataCorp LLC, College Station, TX, USA). The unit of analysis is the individual. Potential confounders will be examined including age, gender, antimalarial use, type of housing, and indicators of socio-economic status at the individual level; malaria intervention use at the household and individual levels, and elevation, village size, and mosquito-habitats (i.e. forest cover, bodies of water) at the village level. We will conduct intent-to-treat analyses. If it is decided that an interim analysis needs to be conducted, the study design will be adjusted for the p-value spent.

### Modeling

Mathematical modeling and simulation analyses have the potential to assist with analysis and interpretation of randomized trials of interventions, particularly if trial results are unexpected [50], as well as estimation of the effectiveness of interventions in dynamic conditions beyond the trial period. There is a long history of mathematical models of malaria transmission, ranging from deterministic to stochastic models, with different levels of complexity [51]. We will develop an epidemiological compartmental model with host-vector interactions to study the impact of a combination of interventions (i.e. ITNs, IRS, and MCs) on transmission dynamics of malaria. The model will build on existing compartmental models of malaria employing the widely used Susceptible-Infected-Recovered (SIR) framework to track host and vector populations and their interactions through contact [52,53]; but, we will extend these models to allow for greater host heterogeneity, where hosts can be grouped according to their level of immunity (i.e. non-immune, semi-immune, immune), availability to mosquitoes (i.e. protected and unprotected), and level of infectiousness to mosquitoes based on level of intervention coverage (i.e. vector control and mass screening and treatment). The extended model will be able to accommodate the effects of several types of interventions and provide a platform to (1) assess the impact of interventions singly or in combination, (2) study the consequences of uneven intervention coverage, and (3) determine the most effective combination of interventions in reducing infection rates toward the goal of malaria elimination in the local setting. For this, parameter values to calibrate the model are either obtained directly from the epidemiological or laboratory data collected from the trial, or are estimated statistically using existing datasets from CSCMi for the study setting or from the published literature [54–60]. The model is coupled with a health economic decision model to assess the economic efficiency and sustainability of the intervention strategies that are found to be effective to inform practice and policy, when supporting data on costs of interventions and malaria illness are available. Modelling will be conducted in STELLA Architect system dynamics modeling tool, version 1.9.4. (isee systems, Lebanon, NH, USA)

## Discussion

By definition of the intervention program criterion, the villages selected by the Odisha state MCP for MCs are located in remote and ‘hard to reach’ parts of the state. Most of the selected villages lack telephone and internet networks and cannot be visited via standard automobile, requiring the study teams to travel long distances on foot. The posed challenges are exacerbated in the months of peak malaria transmission (e.g. monsoon seasons) when rains are frequent and heavy and many of the roadways are inaccessible or washed out entirely. Close coordination of study visits and continued collaboration with the DAMaN teams, the local health centers and personnel including the village ASHAs, and the Odisha state MCP have so far aided greatly in tackling these logistic challenges.

These remote and rural regions of Odisha are largely populated by tribal groups. Some of the study villages in the Keonjhar district are home to the Juang and Munda tribes, two populations that operate largely independent of surrounding communities and are not accustomed to outside contact. Villagers of the stated tribes demonstrated a greater reluctance to participate in the study than observed in other study villages, and many of the individuals who gave initial written consent to participate were not willing to give a blood sample.

### Study status

**Figure 2.**
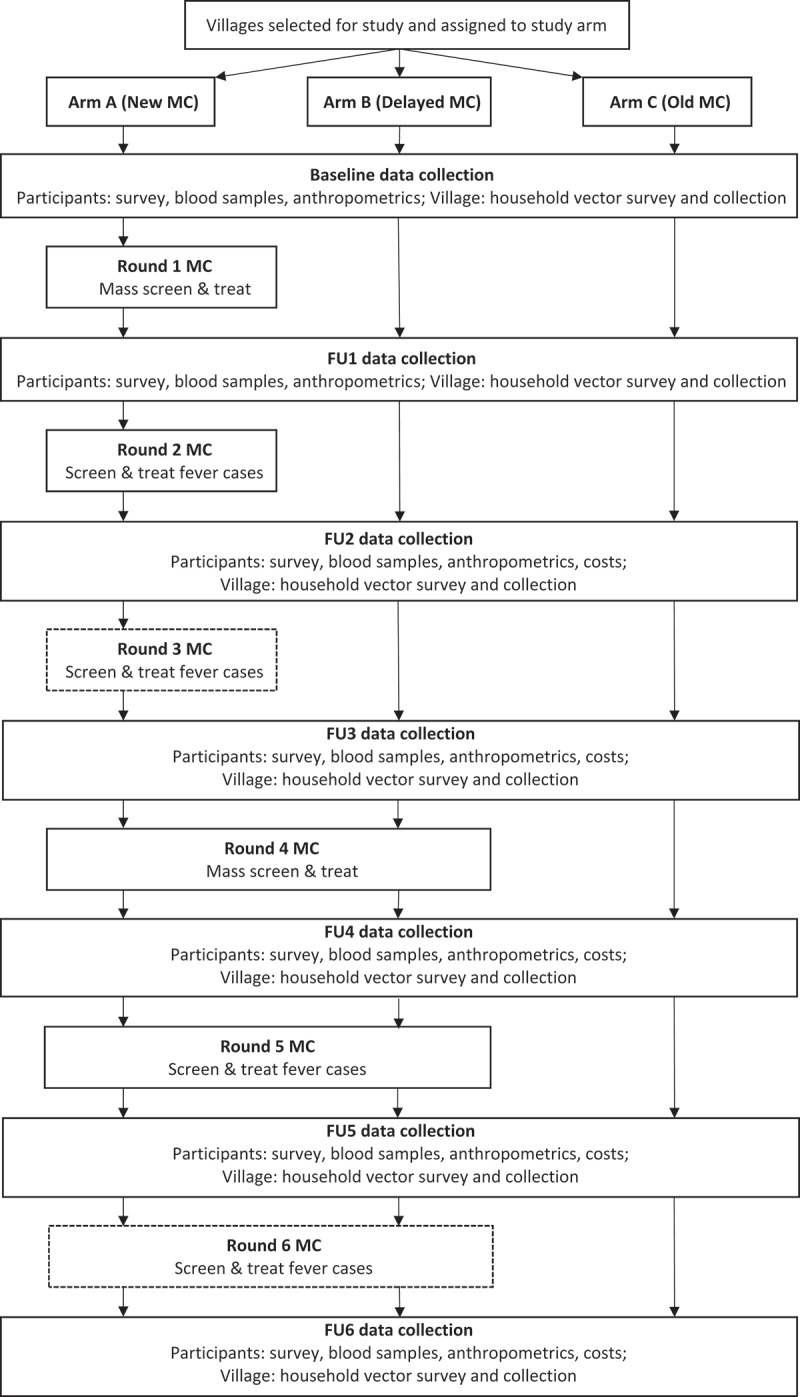
Study flow diagram

On 25 March 2020, the study was temporarily suspended following Prime Minister Narendra Modi’s national lockdown in response to the COVID-19 global pandemic. The national lockdown was imposed in all Indian states with regulations including the closure of all non-essential services and businesses including places of worship, suspension of all education and research operations, suspension of non-essential transportation, prohibition of all social, political, sports and entertainment, and cultural activities, and bans on leaving one’s household. The nationwide lockdown was first extended through 3 May 2020 and subsequently until 8 June 2020. All districts were classified into risk zones (e.g. ‘green’ = 0 reported cases in previous 3 weeks; ‘orange’ = limited number of reported cases; ‘red’ = hotspots) and re-evaluated on a weekly basis. The lockdown was extended through May 31 for all ‘orange’ and ‘red’ zones. The study site districts were ‘orange zones’ at the time of the extension, so study operations remained suspended. The study region began re-opening on 8 June 2020, and data collection for the study resumed with the second follow-up visit during the week of 22 June 2020 (Figure 2). Due to the pandemic, we were only able to collect data from five villages for the first follow-up visit.

During the writing of this manuscript, the study protocol of a complementary project ‘DAMaN Assessment’ was published by colleagues at the Indian Council of Medical Research/Regional Medical Research Centre, Bhubaneswar, India [61].

### Other limitations

There are several important limitations to this study. Villages were not randomized to study arms, which may result in potential selection bias. However, our assignment of villages aimed to ensure that the distribution of villages across the arms was comparable in terms of ecology and terrain type, presence/absence of mining activity, population size, and API. Given the current COVID-19 pandemic, loss-to-follow-up could lead to selection bias as well. With respect to information bias, all self-reported survey measures, including malaria symptoms and diagnoses, may be subject to recall bias. We note that all participants are surveyed prior to receiving the MC screen and treat protocol, but other activities may be occurring including the educational components of the MC intervention package. This could result in possible information bias, resulting in an overestimation of malaria symptoms knowledge in the study sample.

## Summary

Implementation research is intended to combine research approaches and real-life practices to expedite both the general development and delivery of effective public health policies and programs [62]. Here, we apply molecular detection methods, serology, genomics, entomology, costing, and modeling techniques to supplement the ongoing work of the Odisha state MCP with the shared goal of determining the impact of MCs on malaria prevalence, vector populations, and community transmission rates. The MCs represent a major government funded program aimed at combatting a vector-borne disease that poses great health and economic burden to the state. Improved knowledge of MC effectiveness and costing has the potential to improve the program’s delivery, thereby improving both community health and the local economy. The combination of advanced technologies with local expertise and social capital as described here is imperative to tackling global public health problems such as malaria. Our results will be synthesized into a set of recommendations on optimal malaria control strategies and will be presented to the Odisha state MCP.

## Paper context

To address the persistently high rates of malaria in remote and rural regions, the Odisha State (India) Malaria Control Program introduced ‘malaria camps’ to provide education, enhance vector control methods, and perform village-wide screening and treatment. This study protocol describes a cluster-assigned quasi-experimental stepped-wedge study with a pretest-posttest control group design that evaluates if malaria camps reduce the prevalence of malaria, compared to control villages which receive the usual malaria control interventions.
